# Long-term postoperative pneumonia in elderly patients with early gastric cancer

**DOI:** 10.1186/s12893-022-01670-4

**Published:** 2022-06-07

**Authors:** Ayako Kamiya, Tsutomu Hayashi, Ryota Sakon, Kenichi Ishizu, Takeyuki Wada, Sho Otsuki, Yukinori Yamagata, Hitoshi Katai, Takaki Yoshikawa

**Affiliations:** Department of Gastric Surgery, National Cancer Center Hospital, 5-1-1 Tsukiji, Chuo-ku, Tokyo, 104-0045 Japan

**Keywords:** Pneumonia, Gastrectomy, Elderly patients, Sarcopenia

## Abstract

**Background:**

Pneumonia is a major cause of death in the elderly population. Considering body weight loss, muscle loss, and reflux after gastrectomy, elderly patients are considered to be at very high risk for pneumonia, which could decrease overall survival because early gastric cancer is mostly curable only by surgery. We aimed to clarify the incidence of pneumonia in the long-term period after gastrectomy in elderly patients who were diagnosed with early gastric cancer and its risk factors.

**Methods:**

We retrospectively examined patients of > 75 years of age who underwent R0 gastrectomy for gastric cancer and who were diagnosed with T1 disease at National Cancer Center Hospital between 2005 and 2012. Long-term postoperative pneumonia was diagnosed by chest computed tomography every year until 2 years after surgery. The presence of preoperative sarcopenia was assessed using preoperative L3 skeletal muscle index.

**Results:**

167 patients were included in this study. Long-term postoperative pneumonia was observed in 44 (26%) patients. Of the 44 people diagnosed with long-term postoperative pneumonia, 33 were diagnosed in the 1st year and 11 in the 2nd year. 117 patients (70%) were diagnosed with sarcopenia which was significantly frequently found in the patients who developed long-term postoperative pneumonia (91%) than those without (63%). Preoperative sarcopenia was the only independent risk factor in multivariate analysis. Type of gastrectomy was not a significant risk factor.

**Conclusions:**

Long-term postoperative pneumonia was frequently observed in the elderly patients. Preoperative sarcopenia was associated with long-term postoperative pneumonia in elderly patients who underwent curative surgery for gastric cancer. After gastrectomy, long-term special care would be required for elderly patients, especially with sarcopenia.

## Background

In Japan, gastric cancer is characterized by increased rate of early-stage disease and occurrence in elderly patients [1, 2]. Early gastric cancer is only curable by local treatment, and cause of death is mostly other than gastric cancer [3, 4]. In the elderly population, pneumonia is one of the most frequent causes of death [5]. Thus, pneumonia in the long-term period after gastrectomy could decrease overall survival in patients with early gastric cancer, which is a serious threat, especially for elderly patients.

Pneumonia in the elderly population is associated with frailty caused by muscle depletion [6]. On the other hand, many physicians reported that gastrectomy easily causes body weight loss and muscle loss after surgery [7, 8]. Furthermore, total gastrectomy can cause reflux and aspiration which are risk factors for pneumonia [9]. Accordingly, elderly patients are considered to have a very high risk of developing pneumonia in the long-term period after gastrectomy. To separate the term of perioperative pneumonia, we called this pneumonia developing in the long-term period after gastrectomy “long-term postoperative pneumonia (LTPP)” in the present study.

There have been no reports focusing on LTPP in elderly patients. Its incidence and risk factors remain unclear. Physicians may select endoscopic treatment instead of gastrectomy, even in cases that endoscopic treatment is not oncologically justified, if the frequency of LTPP is high or if physicians can identify patients who are at high risk for LTPP.

In this background, we investigated the frequency of LTPP in the elderly patients who received gastrectomy and were diagnosed with stage I gastric cancer and its risk factors.

## Methods

### Patients

Patients were selected from the clinical database of consecutive patients who underwent gastrectomy for gastric cancer at National Cancer Center Hospital from January 2005 to December 2012, based on the following criteria: (1) pathological diagnosis of T1, (2) age ≥ 75 years, (3) R0 resection achieved, and (4) chest computed tomography (CT) performed before surgery and within 2 years after surgery.

### Surgery and follow-up

Surgery was basically determined by the Japanese Gastric Cancer Treatment Guideline version 2 or version 3 depending on the date of the surgery [10, 11]. In short, gastrectomy with D1 or D1 plus dissection was performed for early cancer without adjuvant chemotherapy. The surgical procedure and extent of lymphadenectomy were determined by oncological tumor characteristics regardless of the patient’s age. The postoperative follow-up was as follows: disease recurrence for stage I tumors was evaluated routinely every 6 months during the 1st year and every year thereafter for the next 4 years. The oncological follow-up included physical examinations, blood tests, and CT scans or ultrasonography. Basically, chest to abdominal CT scans were performed routinely every year for at least 5 years after surgery for the purpose of oncological follow-up. When recurrence was suspected, additional imaging studies were performed.

### The diagnostic criteria for long-term postoperative pneumonia

**Fig. 1:**
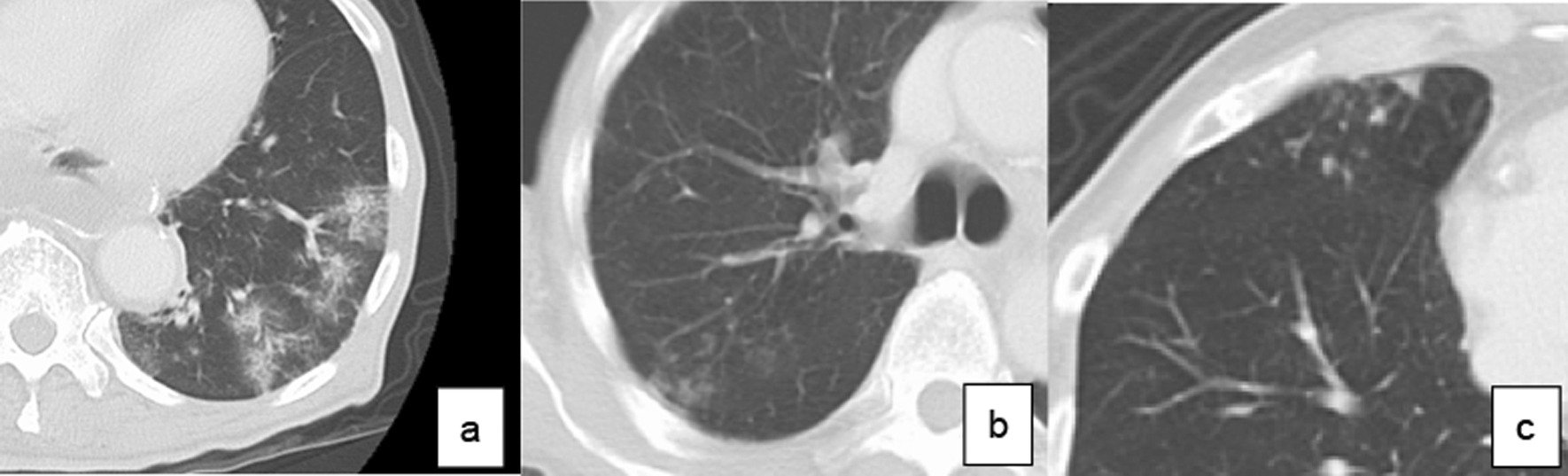
Definition of the diagnosis of pneumonia based on CT **a** consolidation type, **b** reticular type, **c** nodular type

LTPP was diagnosed by chest CT findings performed every year until 2 years after surgery. According to the guidelines for diagnostic imaging of adult community-acquired pneumonia 2007, diagnostic imaging of pneumonia in CT was classified into three types (Fig. 1): consolidation type, reticular type, and nodular type [12]. Those presenting two or more types at the same time were called as mixed types. The diagnosis of LTPP was defined as the new presence of these findings on chest CT compared to CT performed before surgery. CT detected “LTPP”, even in patients who had not shown any active symptoms of pneumonia.

After the certificated radiologists checked the radiological findings, two surgeons evaluated the images for the diagnosis of LTPP. If the diagnosis of the two surgeons differed, the images were evaluated again and final judgments were made. The patients were classified into those with LTPP (P group) and without LTPP (C group).

### Evaluation of preoperative sarcopenia

**Fig. 2:**
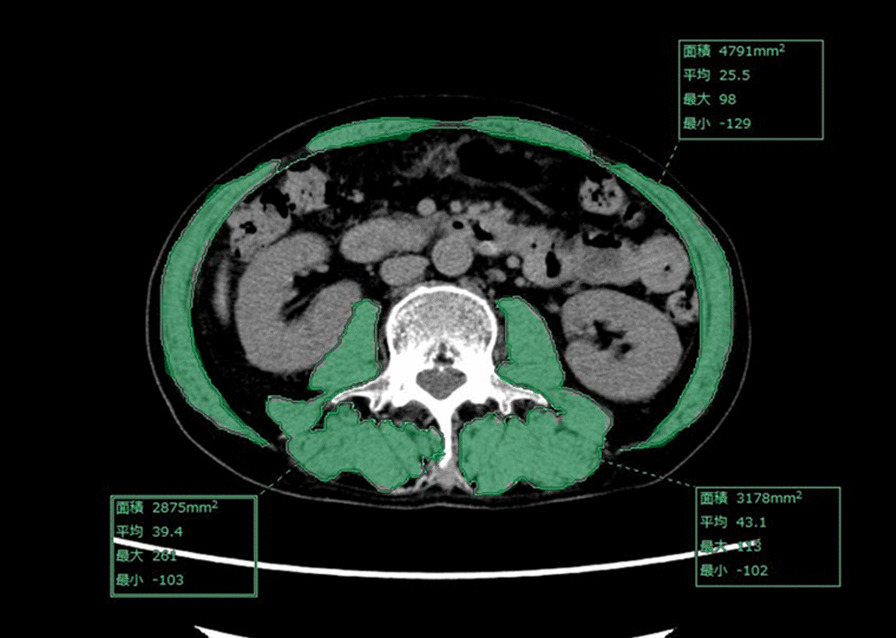
Axial computed tomography slice of the third lumbar vertebra (L3) green areas indicate skeletal muscle

Preoperative sarcopenia was evaluated by measuring the skeletal muscle area by the SYNAPSE VINCENT system based on CT [13]. The evaluated samples were axial slices of the third lumbar vertebrae (L3) [14]. The L3 region contains psoas, paraspinal muscles, and abdominal wall muscles (Fig. 2). The skeletal muscle area in a single abdominal image is reportedly proportional to the whole-body muscle mass [15]. The muscle area normalized by the square of the height (m) is called the L3 skeletal muscle index [SMI, (cm^2^/m^2^)] [16]. We investigated the preoperative L3 SMI in the CT image. Preoperative sarcopenia was defined as an L3 SMI value less than the sex-specific cut-off point of L3 SMI. Each cut-off value of L3 SMI was set at the value where the odds ratio was at its maximum.

### Statistical analysis

SPSS version 15.0 (Statistical Package for the Social Sciences; SPSS, Chicago, IL, USA) was used to perform the statistical analyses. To identify risk factors for LTPP, age, surgical procedure, Charlson score [17], Body Mass Index [BMI, body weight (kg)/height (m)^2^], % vital capacity (%VC) and forced expiratory volume in 1 s (FEV1%) were converted to binary data. Statistical comparisons of the differences in each variable between the C and the P groups were made using the Chi-squared test. Variables were also investigated by a multivariate logistic regression analysis to assess the risk factors associated with LTPP. p values of < 0.05 were considered to indicate statistical significance.

## Results

### Patients demographics

**Fig. 3:**
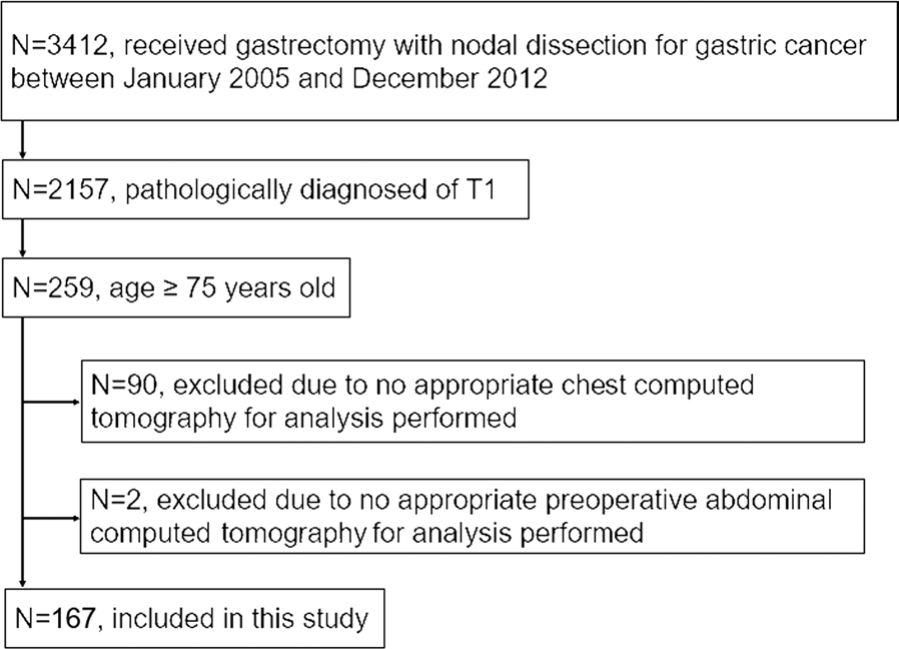
Flow diagram of the present study

Among the 3412 patients who underwent gastrectomy for gastric cancer at National Cancer Center Hospital from 2005 to 2012, a total of 167 patients were included in the present study. Figure 3 shows the consort diagram of the present study. LTPP was found in 44 patients (26%): consolidation type (n = 15), reticular type (n = 12), nodular type (n = 14), and mixed type (n = 3) (Table 1). Of the 44 people diagnosed with LTPP, 33 were diagnosed in the first year and 11 in the second year. The agreement rate in the diagnosis of LTPP was 96% in two surgeons.

**Table 1:** Types of long-term postoperative pneumonia in the study population

	n = 44 (%)^a^
Consolidation type	15 (34)
Reticular type	12 (27)
Nodular type	14 (32)
Mixed type	3 (7)

^a^The number and percentage of patients with long-term postoperative pneumonia

Table 2 summarizes the clinicopathological characteristics of the patients. All patients were diagnosed as pathological Stage I. Surgery, co-morbidity, smoking history, and respiratory function were almost similar between the two groups. Seventy-five patients had a pneumonia shadow before surgery. Among them, 24 patients developed LTPP at another location of the lung until 2 years after surgery. In the present study, the cut-off values of preoperative sarcopenia for males and females were 49.2 and 35.7 cm^2^/m^2^, respectively. By this cut-off, 117 patients (70%) were diagnosed with sarcopenia which was significantly frequently found in the P group (91%) than the C group (63%). During the follow-up period, 1 patient in the C group died of recurrent disease, and 28 patients (12 patients of the C and 16 patients of the P group) died of causes other than gastric cancer.

**Table 2:** Clinicopathological characteristics

Variables	Total N (%)^†^	C group (n = 123) N (%)^†^	P group (n = 44) N (%)^†^
Age (Median, range)	77 (75–87)	78 (75–87)	77 (75–85)
Sex			
Male	109 (65)	74 (60)	35 (80)
Female	58 (35)	49 (40)	9 (20)
Surgical approach			
Open	153 (92)	111 (90)	42 (95)
Laparoscopy	14 (8)	12 (10)	2 (5)
Surgical procedure			
Distal gastrectomy	80 (48)	60 (49)	20 (46)
Pylorus preserving gastrectomy	45 (27)	33 (27)	12 (27)
Total gastrectomy	28 (17)	20 (16)	8 (18)
Proximal gastrectomy	14 (8)	10 (8)	4 (9)
BMI (Median, range)*	22.6 (16.5–32.2)	22.2 (16.7–32.2)	23.0 (16.5–30.5)
GNRI (Median, range)**	104.5 (77.1–128.0)	104.5 (77.1–125.3)	104.3 (89.9–128.0)
Charlson Score			
0	93 (55)	70 (57)	23 (52)
1	34 (20)	25 (20)	9 (21)
2	25 (15)	17 (14)	8 (18)
3	9 (5)	6 (4)	3 (7)
4	2 (1)	2 (2)	0 (0)
5	0 (0)	0 (0)	0 (0)
6	3 (2)	2 (2)	1 (2)
7	1 (1)	1 (1)	0 (0)
Current smoking history			
+	73 (44)	53 (43)	20 (45)
−	94 (56)	70 (57)	24 (55)
%VC (Median, range)	103 (53–175)	104.5 (53–175)	100 (69–133)
FEV1% (Median, range)	73 (31–105)	74 (34–92)	72 (31–105)
Sliding Hernia			
+	49 (29)	36 (29)	13 (30)
−	118 (71)	87 (71)	31 (70)
Preoperative pneumonia			
+	76 (46)	52 (42)	24 (55)
−	91 (54)	71 (58)	20 (45)
Preoperative sarcopenia			
+	117 (70)	77 (63)	40 (91)
−	50 (30)	46 (37)	4 (9)
Pathological TNM stage			
T1aN0	70(42)	52 (42)	18 (41)
T1aN1	1 (1)	0(0)	1(2)
T1bN0	84 (50)	62 (51)	22 (50)
T1bN1	12 (7)	9 (7)	3 (7)

%VC: Vital capacity; FEV1%: Forced expiratory volume in 1 s

^†^Number and percentage of patients in each category in each group (except age, BMI, GNRI, %VC and FEV1%)

BMI*: Body Mass Index = (body weight (kg)/height (m^2^))

GMRI**: Geriatric Nutritional Risk Index = 14.89 × Alb (g/dl) + 41.7 × (body weight (kg)/ideal body weight (kg))

### Risk factors for long-term postoperative pneumonia in the univariate and multivariate analyses

To identify risk factors for LTPP, variables were converted to binary data (Table 3). The surgical procedures were classified into two groups based on whether or not the cardia was preserved (distal gastrectomy and pylorus preserving gastrectomy), which would be related to reflux symptoms. In our study population, 75% of patients received cardia preserving gastrectomy. Among the variables included in the univariate analyses, male sex and preoperative sarcopenia were the factors that showed a significant difference between the C and P groups (p = 0.026 and p = 0.000, respectively). In the multivariate analysis, preoperative sarcopenia was the only independent risk factor for LTPP (p = 0.003).

**Table 3:** Risk factors for long-term postoperative pneumonia in the univariate and multivariate analyses

Variables	Total N (%)^†^	C group N (%)^†^	P group N (%)^†^	Univariate analysis	Multivariate analysis		
				*p*^††^	Odds ratio	*p*^†††^	95% confidence interval
Age				0.440	1.290	0.529	0.580–2.890
< 80	118 (71)	89 (72)	29 (66)				
80≤	49 (29)	34 (28)	15 (34)				
Sex				0.026	2.110	0.090	0.890–5.020
Male	109 (65)	74 (60)	35 (80)				
Female	58 (35)	49 (40)	9 (20)				
Surgical procedure				0.691			
DG + PPG	125 (75)	93 (76)	32 (73)				
TG + PG	42 (25)	30 (24)	12 (27)				
Charlson score				1.000			
< 3	152 (91)	112 (91)	40 (91)				
3≤	15 (9)	11 (9)	4 (9)				
BMI				0.602			
< 22.5	82 (49)	62 (50)	20 (45)				
22.5≤	85 (51)	61 (50)	24 (55)				
Current smoking history				0.860			
+	73 (44)	53 (43)	20 (45)				
−	94 (56)	70 (57)	24 (55)				
%VC				1.000			
< 80	13 (8)	10 (8)	3 (7)				
80≤	152 (92)	112 (92)	40 (93)				
FEV1%				0.703			
< 70	52 (32)	37 (30)	15 (35)				
70≤	113 (68)	85 (70)	28 (65)				
Sliding Hernia				1.000			
+	49 (29)	36 (29)	13 (30)				
−	118 (71)	87 (71)	31 (70)				
Preoperative pneumonia				0.217	1.540	0.254	0.733–3.240
+	75 (45)	52 (42)	24 (55)				
−	91 (55)	71 (58)	20 (45)				
Preoperative sarcopenia				0.000	5.380	0.003	1.770–16.30
+	117 (70)	77 (63)	40 (91)				
−	50 (30)	46 (37)	4 (9)				

DG: distal gastrectomy; PPG: pylorus preserving gastrectomy; TG: total gastrectomy; PG: proximal gastrectomy; BMI: Body Mass Index = body weight (kg)/height (m)^2^; %VC: Vital capacity; FEV1%: Forced expiratory volume in 1 s

^†^The number and percentage of patients in each category in each group

^††^Variables were analyzed by a Chi-squared test

^†††^Variables were analyzed by a logistic regression analysis

## Discussion

In the present study, we called the pneumonia shadow found in the long-term period after gastrectomy “LTPP” and examined its incidence and risk factors in elderly patients who received curative gastrectomy and who were diagnosed with early gastric cancer. We firstly clarified that more than one quarter of the elderly patients developed LTPP within 2 years after gastrectomy. This high incidence must impact the decision on whether the patient should receive surgery or not. Moreover, preoperative sarcopenia was the only significant risk factor for LTPP. After gastrectomy, special care through the long-term period is required for elderly patients, especially with sarcopenia.

In this study, LTPP was observed in 26% of elderly patients. There are no reports on the frequency of LTPP in any age group. On the other hand, the incidence of hospitalized community-acquired pneumonia in the elderly was reported to be approximately 2% in the United States [18]. Kaplan et al. also reported that its incidence was 8.4 per 1000 in individuals of aged 65–69 years of age and 48.5 per 1000 in individuals of ≥ 90 years of age [19]. Although our results showed a higher incidence in comparison to the above reports, the incidence in our study would be overestimated. In this study, LTPP was diagnosed based on chest CT images, by which even minimal changes of pneumonia shadow can be detected [20]. Therefore, this study included cases of asymptomatic pneumonia or pneumonia that did not require medical treatment. Even if the incidence in our study was overestimated, the very high incidence of LTPP is a serious issue, because early gastric cancer is mostly curable and pneumonia is a major cause of death in the elderly population.

Aging is associated with loss of muscle mass and physical function, that often leads to progressive disability [21]. The amount of muscle begins to decrease after the age of 50, and approximately 50% are lost until the age of 80 [22]. Sarcopenia has been defined as the loss of muscle mass and strength that occurs with aging [23, 24]. In the present study, sarcopenia was observed in 70% of the elderly patients who had early gastric cancer, that seemed to be higher than general elderly people. Approximately 22% of men and women aged 75–79 years old and 32.4% of men and 47.7% of women aged 80 years and older [25]. As definition of sarcopenia would be different, it is unclear whether elderly patients who developed gastric cancer have high risk of sarcopenia.

Several studies have shown that gastric cancer patients with sarcopenia at the time of surgery experienced worse long-term outcomes than non-sarcopenic patients [26–30]. Pneumonia in the elderly population is associated with frailty caused by muscle depletion [6]. Altuna-Venegas et al. reported that sarcopenia was a risk factor for the onset of pneumonia [31]. In the present study, more than 90% of the elderly patients who developed LTPP had sarcopenia. Thus, preoperative sarcopenia would have high risk of LTPP for elderly patients who received gastrectomy for gastric cancer. Moreover, gastrectomy itself frequently induces loss of body weight and muscle, which would be severe especially in patients with preoperative sarcopenia. Thus, progressive sarcopenia and frailty would closely associated with LTPP in the elderly patients who received gastrectomy.

Originally, diagnosis of sarcopenia required low muscle mass plus either low muscle strength or low physical performance and the diagnosis criteria was defined for aging patients [23, 24]. However, many studies have performed CT-based body composition measurement and muscle area from CT has been used to define sarcopenia. Since CT is taken for staging in almost all cases, the indirect CT method is readily applicable. It is of note that the cutoff value to define sarcopenia differs in each study and has not yet been standardized [32]. One reason is that differences in the nutritional status among cancer patients are actually very large. Another reason in sarcopenia studies is the difficulty of obtaining normal CT values from areas of muscle mass in a healthy cohort. In the present study, the cut-off values of preoperative sarcopenia for males and females were 49.2 and 35.7 cm^2^/m^2^, respectively. They were similar to the previous reports examining the preoperative sarcopenia of gastric cancer patients, in which the cut-off values for males and females were 40.8–55 and 34.9–41 cm^2^/m^2^, respectively [26–29].

Previously, surgical procedure, comorbidity, and preoperative respiratory function have been reported as risk factors for perioperative pneumonia [33–37], while age, gender and comorbidity have been reported as risk factors for community-acquired pneumonia [19, 38, 39]. In this study, age, comorbidity, the preoperative respiratory function, and the type of gastrectomy were not significant risk factors for LTPP. Thus, risk factors for LTPP after gastrectomy might be different from those of perioperative pneumonia or community-acquired pneumonia. On the other hand, the type of gastrectomy was not associated with LTPP. Notably, the risk of LTPP was similar irrespective of the loss of the cardia, suggesting that reflux was not a major determinant for LTPP. Previously, Marumo et al. reported that reflux and aspiration after total gastrectomy was related to pneumonia [9]. On the other hand, Jackson et al. reported that a poor functional status and recent weight loss were major risk factors for community-acquired pneumonia in the elderly [38]. Gastrectomy easily causes body weight loss, and muscle loss which would decrease activity of daily life, especially in elderly patients. Thus, it would be possible that gastrectomy itself is a risk factor for pneumonia in elderly patients.

The present study was associated with some limitations. First, this was a retrospective single-center study. Our results should be further validated in a multicenter study. Second, the frequency of LTPP may be underestimated because our hospital specializes in oncology and often performs surgery for patients with few comorbidities. In the community hospital setting, where surgeons operate on elderly patients with severe comorbidities, LTPP would be more frequent because such patients would be sarcopenic.

## Conclusions

LTPP was frequently observed in elderly patients regardless of the type of gastrectomy. Preoperative sarcopenia was associated with LTPP in elderly patients who underwent curative surgery for gastric cancer. We expect our findings to help inform the better treatment strategies for elderly gastric cancer patients. After gastrectomy, special care, such as nutritional support, respiratory rehabilitation, oral care and swallowing rehabilitation, through the long-term period is required for elderly patients, especially with sarcopenia.

## Data Availability

All data analyzed during this study are included in this published article.

## Abbreviations

LTPP: Long-term postoperative pneumonia
CT: Computed tomography
SMI: Skeletal muscle index
BMI: Body Mass Index
%VC: % Vital capacity
FEV1%: Forced expiratory volume in 1 s
